# Mitral valve infective endocarditis with spread of infection to the pulmonary valve via coronary artery pulmonary artery fistula: a case report

**DOI:** 10.1186/s44215-025-00200-x

**Published:** 2025-03-04

**Authors:** Hiroharu Shinjo, Shoichi Takahashi

**Affiliations:** Department of Cardiovascular Surgery, Hoshi General Hospital, 159-1, Mukaigawara, Koriyama, Fukushima, Japan

**Keywords:** Infective endocarditis, Lung abscess, Coronary artery pulmonary artery fistula, Pulmonary valve replacement

## Abstract

**Background:**

In cases of left-sided infective endocarditis (IE) complicated by one or more lung abscesses, close examination should be performed with the additional presence of right-sided IE in mind. Pulmonary valve IE may occur via a coronary artery pulmonary artery fistula (CAPAF) even in the absence of vegetation at the tricuspid valve.

**Case presentation:**

A 76-year-old male was admitted to his local hospital with back pain and weight loss that had started 4 months previously. He was diagnosed with vertebral osteomyelitis, and antibiotic therapy was started. Subsequently, echocardiography revealed mobile vegetation at the mitral valve, and computed tomography (CT) showed multiple lung abscesses. The patient was then transferred to our hospital for urgent surgical intervention. Additional echocardiography revealed no visible vegetation at the tricuspid valve but did show thickening and moderate regurgitation of the pulmonary valve. These results indicated the presence of pulmonary valve IE. In addition, coronary CT angiography revealed CAPAF and intraoperative findings showed vegetation on the pulmonary valve. Therefore, mitral valve replacement (MVR), pulmonary valve replacement (RVR), and CAPAF closure were performed.

**Conclusions:**

The present report is thought-provoking to describe the diagnosis of and surgical planning for IE. Firstly, when left-sided IE is complicated by lung abscess, a detailed evaluation of the right heart system and the potential for a left-to-right shunt should be performed, keeping in mind the possible presence of right-sided IE. Secondly, even if there is no vegetation at the tricuspid valve, there may be vegetation at the pulmonary valve, in which case an extracardiac left-to-right shunt that does not pass through the tricuspid valve may be present. CAPAF is a rare anomaly, but it causes pulmonary valve IE, which requires PVR.

## Background

When mitral valve IE with mobile and/or large vegetation is detected, urgent surgical intervention is required to avoid a potential embolism. However, care must be taken, because if the decision to perform intervention is made too quickly, other important diagnoses may be missed, thereby negatively affecting clinical management. If preoperative examination reveals a lung abscess, it is important to determine the presence of right-sided IE, because a lung abscess may be caused by a septic pulmonary embolism or a pulmonary embolism caused by the shedding of the bacterial mass.

Even in the absence of obvious tricuspid valve vegetation, there is the possibility of the presence of a left-to-right shunt or, although rare, pulmonary valve IE. The shunt can be intracardiac or extracardiac, especially in the case of CAPAF, which is a rare etiology.

In the present case, we experienced a case of mitral valve IE with lung abscesses. Therefore, a detailed preoperative examination was performed, revealing thickening and regurgitation of the pulmonary valve, as well as the presence of a CAPAF.

## Case presentation

A 76-year-old male was admitted to his local hospital with back pain and weight loss that had started 4 months previously and was diagnosed with vertebral osteomyelitis. Two separate blood cultures were both positive for streptococcus gordonii, and the patient was started on antibiotic therapy with Ampicillin/Sulbactam. Ten days after admission, echocardiography revealed a thickened mitral valve leaflet with a 15-mm mobile vegetation, as well as moderate mitral regurgitation. No obvious vegetation was observed on the other valves. After antibiotic therapy was initialed, the patient’s fever remained higher than 38 ℃, and he had a persistently high inflammatory response. He required urgent surgical intervention due to the mobile vegetation and refractory infection, although no heart failure symptoms were observed. As a result, the patient was transferred to our hospital 12 days after initial hospitalization.

**Fig. 1:**
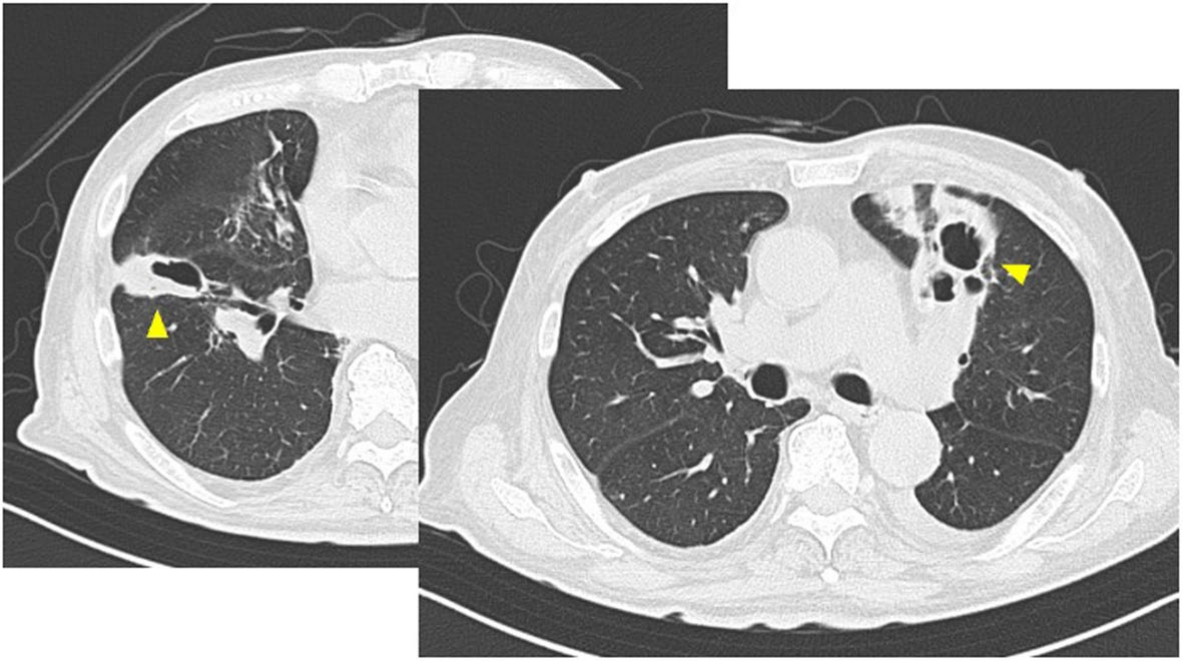
Preoperative chest CT shows multiple bilateral lung abscesses (arrowheads)

His medical history included hypertension and hyperlipidemia, with a height of 169.0 cm and a weight of 67.0 kg (body surface area 1.769 m^2^). His vital findings on admission were: body temperature 38.1 ℃; blood pressure 125/78 mmHg; pulse 85 bpm; and SpO2 94%(1 L/nasal). The patient was not in cardiogenic shock. Laboratory tests on admission showed a white blood cell count of 9400/µl, C-reactive protein of 6.36 mg/dl, and an elevated brain natriuretic peptide of 251.8 pg/ml. There were no other findings of note except for low nutritional status. CT performed at the previous hospital revealed multiple lung abscesses in the bilateral lungs (Fig. 1).

**Fig. 2:**
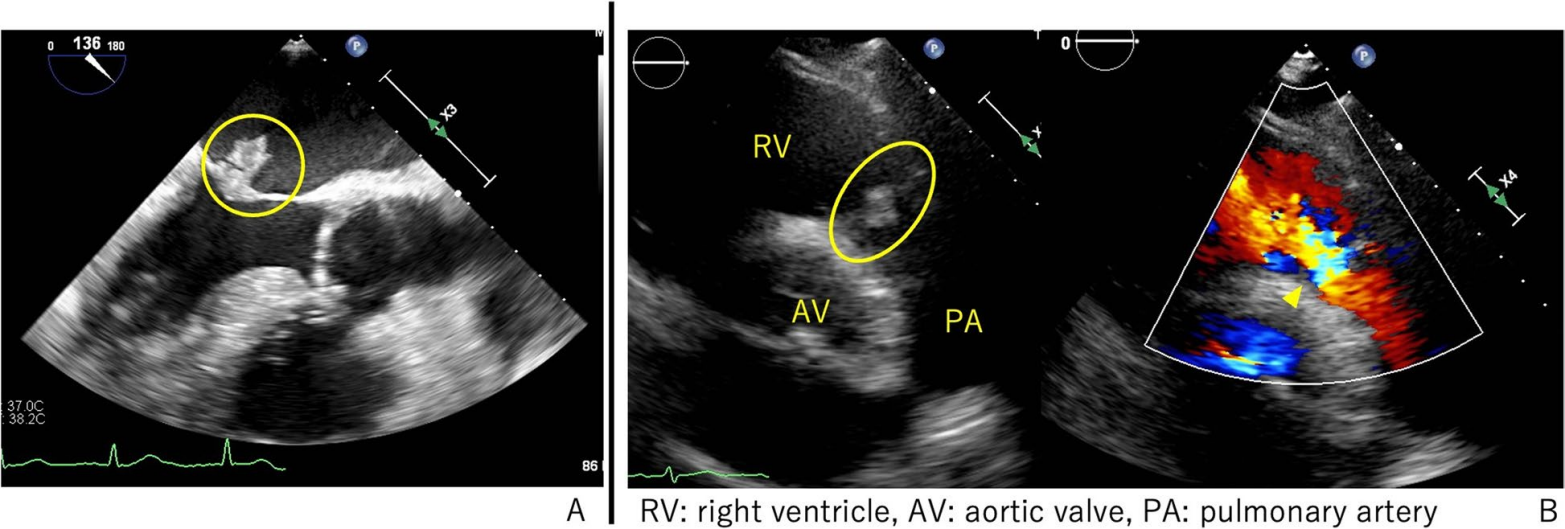
Preoperative echocardiography. **A** There was 10 mm vegetation adherent to the mitral valve (circle). **B** There was a thickening of pulmonary valve (oval) and moderate pulmonary valve regurgitation (arrowhead)

We performed a second echocardiography, which revealed good cardiac function, as well as a 10-mm mobile vegetation on the anterior mitral leaflet, as well as mild mitral regurgitation (Fig. 2A). The pulmonary valve was found to have thickened leaflets and moderate regurgitation (Fig. 2B). There was no obvious intracardiac left-to-right shunt.

**Fig. 3:**
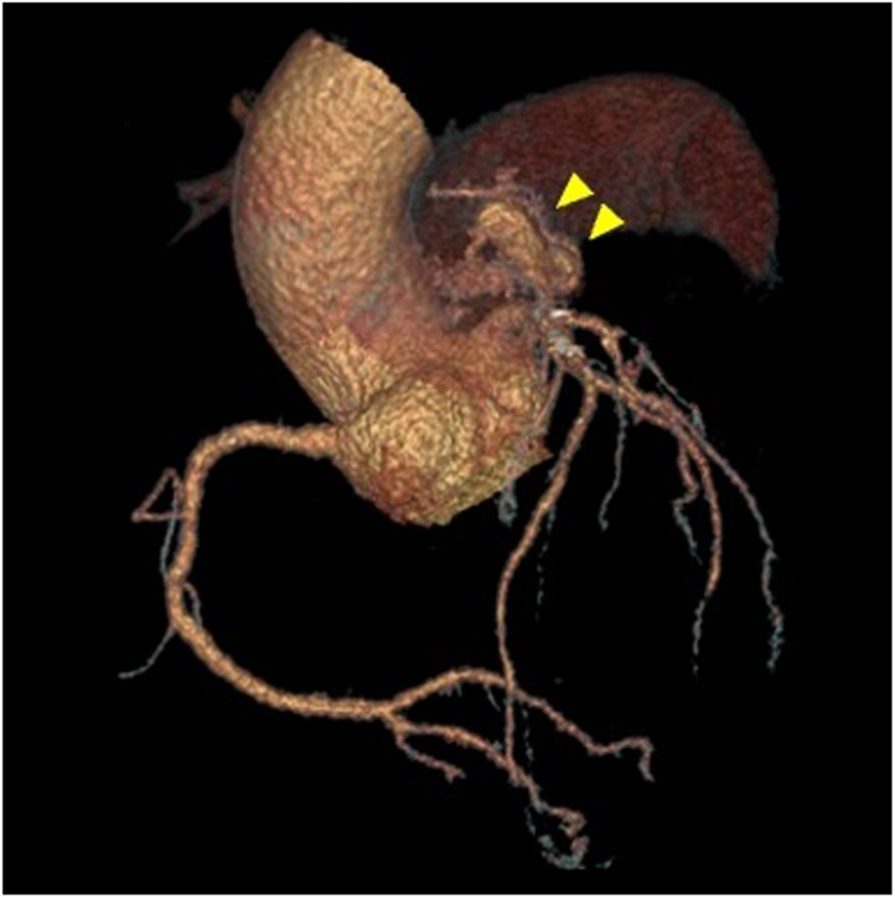
Preoperative coronary CT angiography of CAPAF. The fistula was tortuous with a partly dilatated tract from the left anterior descending artery into the main pulmonary artery (double arrowheads)

Further, coronary CT angiography revealed a CAPAF from the left anterior descending artery to the main pulmonary artery (Fig. 3). A small abscess was observed in the iliopsoas muscle, but there were no obvious embolic findings in the other organs. The preoperative risk score (Japan SCORE II) revealed a 30-day mortality of 5.4% and a major complication rate of 17.8%.

**Fig. 4:**
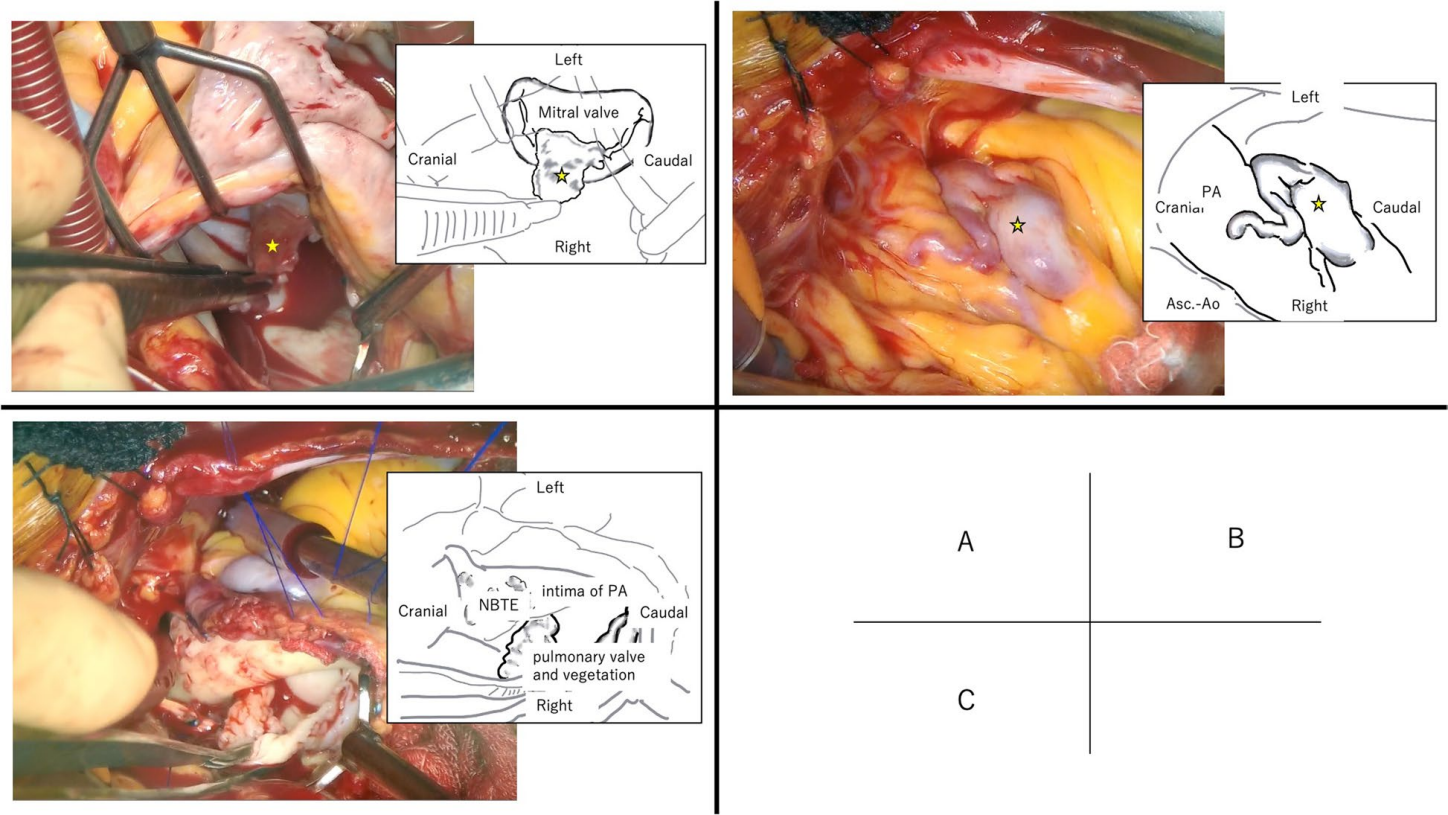
Operative findings: A vegetation adherent to the mitral anterior leaflet (star). There were other small vegetations adherent to the mitral posterior leaflet, not visible in this figure. B CAPAF on the main pulmonary artery (star). C Nonbacterial thrombotic endocarditis (NBTE) attached to the intima of the pulmonary artery and vegetation adherent to the pulmonary valve

MVR, PVR, and closure of the fistula were performed 3 days after transfer to our hospital. No vegetation was observed at the tricuspid valve, and the right atrial septum was intact. A right-sided left atriotomy was performed, and when the mitral valve was exposed, vegetation was observed on A2 (Fig. 4A). The vegetation was excised and a Cavitron ultrasonic surgical aspirator (CUSA) (Integra Japan, Corp., Tokyo, Japan) was used to excise the small vegetation in the clear zone, resulting in a large defect in the coaptation zone. An additional vegetation attached to the posterior leaflet was also excised. Since mitral valve repair was unfeasible, MVR with a 27-mm ATS (ATS Medical, Inc., Minneapolis, USA) was performed.

The CAPAF covered the anterior surface of the pulmonary artery (Fig. 4B). When viewing the lumen of the pulmonary artery, a fistula orifice was identified on the left side of the incision, and small vegetations were diffusely attached to the intima, extending around the pulmonary valve (Fig. 4C). No patent ductus arteriosus was observed. The fistula orifice was closed directly, and the small intimal vegetations were excised using CUSA. The pulmonary valve was replaced with a 29-mm MITRIS RESILIA mitral valve (Edwards Lifesciences, Irvine, CA) in the opposite direction, and the pulmonary artery was repaired using a bovine pericardial patch. The cardiac arrest time was 216 min and the extracorporeal circulation time was 287 min.

The patient was extubated on postoperative day (POD) 2 and discharged from the intensive care unit on POD6. Grocott staining revealed bacterial masses in both the mitral and pulmonary valves. Antibiotics were continued until POD49. Postoperative echocardiography performed on POD25 revealed that both the mitral and pulmonary valve prostheses had sufficient valve function. Following rehabilitation, the patient was discharged on POD77.

## Discussion and conclusions

In the present case, we found that when left-sided IE is complicated by one or more lung abscesses, a thorough examination should be performed with the presence of right-sided IE in mind. We also determined that the presence of a CAPAF as a cause of right-sided IE should also be taken into account, and PVR should also be performed.

Yuan et al. [1] reported a frequency of lung abscess formation in 3.1% of cases of right-sided IE. Pulmonary embolism is a common complication of right-sided IE, even if it does not develop into a lung abscess. Bamford et al. [2] reported a case of lung abscess that was refractory to treatment, in which echocardiography revealed IE in the pulmonary valve, necessitating PVR. Their findings highlighted the importance of assessment of the right heart system. The Japanese guidelines also state that careful assessment of the right heart system is recommended in cases of pulmonary embolism [3].

In cases of IE of the right heart system, vegetations are most commonly attached to the tricuspid valve, but they may be attached to the pulmonary valve. Pulmonary valve IE accounts for 1.5–2% of infective endocarditis cases, and isolated involvement of the pulmonary valve is rare [4]. Therefore, the absence of vegetation on the tricuspid valve does not necessarily exclude right-sided IE. In the present case, the results of the initial examination that there was no vegetation on the tricuspid valve, and we originally determined that intervention was only required for the mitral valve. However, due to the presence of a lung abscess on preoperative CT, echocardiography was performed again to assess for IE in the right heart system revealing thickening and moderate regurgitation of the pulmonary valve. As a result, pulmonary artery valve IE was detected, and a more appropriate treatment strategy was developed.

On the other hand, as a possible cause of lung abscess, it should be mentioned that right-sided IE is not the only possibility. A left-to-right shunt due to congenital heart disease, such as ventricular septal defect, is also possible. It should be also kept in mind that a left-to-right shunt may not only be intracardiac but also extracardiac. However, as an extracardiac left-to-right shunt, CAPAF is rare. Coronary arteriovenous fistulae (CAVF), including CAPAF, are found in 0.1–0.2% of all patients undergoing coronary angiography [5]. CAVF can present with several comorbidities, sometimes resulting in endocarditis [6]. Although the occurrence of IE in CAVF is reported to be 4% [7], its occurrence in CAPAF specifically has not been reported. The reported cases of IE in CAVF were caused by IE in the tricuspid valve via the right atrium [8], and to the best of our knowledge, there have been no reports of IE in the pulmonary valve only. We suspect that this is because 2/3 of the CAVF returns to the right atrium or right ventricle [6], and the tricuspid valve is more frequently affected by the IE in the CAVF than the pulmonary artery valve. In addition, IE has been reported to occur in 2.3% of adult congenital heart disease [9], including intracardiac shunts, and when the frequency of CAVF is taken into consideration, the possibility of IE in CAPAF is extremely rare. Contrast-enhanced CT angiography is a useful modality for detecting CAVF. In addition, intraoperative findings revealed small vegetation caused by intimal inflammation on the contralateral intima of the CAPAF orifice. This corresponds to the area of abnormal perfusion of CAPAF, and it is known as nonbacterial thrombotic endocarditis (NBTE) [3]. It is thought that NBTE is a precursor stage of IE, in which bacteria adhere to and grow in this area, forming vegetation. In the present case, abnormal blood flow due to CAPAF was considered to have caused pulmonary valve IE.

In conclusion, when cases of left-sided IE are complicated by one or more lung abscesses, we should consider the presence of a left-to-right shunt as well as right-sided IE, and perform a detailed right-heart evaluation. In some cases, there may be no vegetation on the tricuspid valve, with vegetation adhering only to the pulmonary valve. In such cases, an extracardiac left-to-right shunt that does not involve the tricuspid valve may be present. CAPAF is a rare condition, but it may cause pulmonary valve IE, necessitating concomitant PVR.

## Data Availability

Not applicable.

## Abbreviations

IE: Infective endocarditis
CAPAF: Coronary artery pulmonary artery fistula
CT: Computed tomography
MVR: Mitral valve replacement
PVR: Pulmonary valve replacement
CUSA: Cavitron ultrasonic surgical aspirator
POD: Post-operative day
CAVF: Coronary arteriovenous fistula
NBTE: Nonbacterial thrombotic endocarditis
